# Development and Validation of a Post-Operative Non-Union Risk Score for Subtrochanteric Femur Fractures

**DOI:** 10.3390/jcm10235632

**Published:** 2021-11-29

**Authors:** Michalis Panteli, James S. H. Vun, Robert M. West, Anthony J. Howard, Ippokratis Pountos, Peter V. Giannoudis

**Affiliations:** 1 Academic Department of Trauma & Orthopaedics, School of Medicine, University of Leeds, Leeds LS2 9LU, UK; j.vun@nhs.net (J.S.H.V.); anthonyjhoward@aol.com (A.J.H.); hippoun@gmail.com (I.P.); pgiannoudi@aol.com (P.V.G.); 2 Leeds Institute of Rheumatic and Musculoskeletal Medicine, University of Leeds, Leeds LS2 9LU, UK; 3 Leeds Orthopaedic & Trauma Sciences, Leeds General Infirmary, University of Leeds, Leeds LS2 9LU, UK; 4 Leeds Institute of Health Sciences, University of Leeds, Leeds LS2 9LU, UK; R.M.West@leeds.ac.uk; 5 NIHR Leeds Biomedical Research Unit, Chapel Allerton Hospital, Leeds LS2 9LU, UK

**Keywords:** non-union, subtrochanteric, femur, scoring system, risk factors

## Abstract

Background: Our objective was to develop and validate a predictive model for non-union following a subtrochanteric fracture of the femur. Methods: Following institutional board approval, 316 consecutive patients presenting to our institution (84 non-unions) who fulfilled the inclusion criteria were retrospectively identified. To identify potential unadjusted associations with progression to non-union, simple logistic regression models were used, followed by a revised adjusted model of multiple logistic regression. Results: Having established the risk factors for non-union, the coefficients were used to produce a risk score for predicting non-union. To identify the high-risk patients in the early post-operative period, self-dynamisation was excluded. The revised scoring system was the sum of the following: diabetes (6); deep wound infection (35); simple or severe comminution (13); presence of an atypical fracture (14); lateral cortex gap size ≥5 mm (11), varus malreduction (5–10 degrees) (9); varus malreduction (>10 degrees) (20). On the ROC (receiver operating characteristic) curve, the area under the curve (0.790) demonstrated very good discriminatory capability of the scoring system, with good calibration (Hosmer–Lemeshow test; *p* = 0.291). Moreover, 5-fold cross validation confirmed good fit of the model and internal validity (accuracy 0.806; Kappa 0.416). The cut-point determined by Youden’s formula was calculated as 18. Conclusion: This study demonstrates that the risk of non-union can be reliably estimated in patients presenting with a subtrochanteric fracture, from the immediate post-operative period. The resulting non-union risk score can be used not only to identify the high-risk patients early, offering them appropriate consultation and in some cases surgical intervention, but also informs surgeons of the modifiable surgery related factors that contribute to this risk.

## 1. Introduction

Subtrochanteric fractures represent a subset of proximal femoral fractures, encountered between the flare of the lesser trochanter and 5 cm distal to it [1,2]. Because of the high concentration of stresses and the vulnerable blood supply of this region, they are challenging to treat and are associated with a high incidence of complications, with their re-operation rate being reported as high as 4.7% [3]. As intramedullary (IM) nailing offers a biomechanical advantage, including a shorter lever arm of the fixation, a better load sharing and less bending movement across the fracture site and the implant, it remains the ‘gold standard’ of treatment [4,5,6].

A number of patient characteristics such as poor bone stock [7], presence of diabetes [8,9], smoking [8,9], and steroid intake [8,9] have been identified by expert clinical opinion and background literature as potential risk factors for non-union. Moreover, characteristics from the primary surgery such as adequacy of reduction [10], residual gap in the medial surface of the femur in the region of the lesser trochanter [10], need for open reduction [10], varus malalignment (defined as angulation of more than 10° at the fracture site in the femoral shaft) [11,12], tip-apex distance (TAD) [13], and the entry point to the femoral canal [14,15,16,17] have also been reported as potential risk factors. 

Our objective was to develop and validate a predictive model for non-union following a subtrochanteric fracture of the femur. 

## 2. Materials and Methods

### 2.1. Data Sources

Following institutional review board (IRB) approval, a retrospective analysis of all consecutive eligible patients presenting to a Level I Trauma Centre over an eight-year period (January 2009–December 2016) was conducted. Eligibility criteria included skeletally mature patients presenting with a subtrochanteric fracture [1], subsequently managed with a long IM nail. Incomplete fractures, prophylactic nailing for pathological lesions, patients having their primary operations in other institutions, and patients not followed-up until complete clinical and radiological union were excluded from further analysis. In the cases of bilateral fractures on the same patient (at the same or a subsequent episode), only the side operated on first was included in the analysis to ensure all the observations were independent.

Basic demographic characteristics, medical co-morbidities, social history, medications, injury characteristics, biochemical and microbiology investigations, operation details, complications and outcomes were collected and analysed (individual parameters examined are found in Table 1). For the analysis of the fracture and evaluation of adequacy of reduction/fixation, several radiographic measurements were performed on immediate post-operative radiographs as well as follow-up radiographs, in an independent, blinded method, by two of the authors (MP and JV). These included fracture characteristics (Russell Taylor classification [18,19], number of fragments, presence of atypical [20] or pathological features (fractures at site of bony metastasis), involvement of the lesser or greater trochanter), fracture gap in each cortex on the anteroposterior and lateral radiographic views, and restoration of neck shaft angle (compared to the contralateral hip or same hip if pre-injury radiographs were available), TAD, method of distal locking, and position of the tip of the nail mainly in relation to the anterior cortex.

Atrophic non-unions were defined as incomplete fracture healing within nine months following injury, along with absence of progressive signs of healing (callus) on serial radiographs over the course of three consecutive months [21]. Hypertrophic non-unions on the other hand were defined as incomplete fracture healing within nine months following injury, with excessive callus formation and a visible fracture line on serial radiographs, associated with pain at the fracture site. Finally, septic non-unions were defined as non-unions associated with an infection at the fracture site. The diagnosis of infection was based on the presence of clinical signs of infection, increased inflammatory markers (CRP and WCC) and positive microbiology cultures from tissue from the non-union site obtained during revision surgery [22]. Regarding comminution at the fracture site, presence of two fracture fragments was considered as simple comminution, three fragments as moderate comminution and four fragments or more as severe comminution.

### 2.2. Statistical Analysis

The computing environment R (R version 3.6.0) was used for the statistical analysis [23]. Demographic data were presented as count (percentage) or as mean ± SD. Following stratification by progression to a non-union, parametric data were analysed using a Welch unpaired independent t-test, whilst Pearson’s chi square test was utilised for the analysis of count data. To identify potential unadjusted associations with progression to non-union, a simple logistic regression model was used. A revised adjusted model of multiple logistic regression to predict progression to non-union was then used, removing covariates in a stepwise fashion according to their likelihood-ratio chi-square *p*-value (*p*-value of <0.10). 

For the development of the non-union scoring system, all factors identified by the logistic regression model were considered. The weight of each variable was then used to create a point scoring system, using the coefficients. More specifically, the highest potential score was given the score of 100, and the remaining points were assigned according to that, with rounding to the nearest point. We did not include any interaction terms, therefore the risk score for each patient was calculated by the sum of the individual variables. Receiver-operator characteristic (ROC) analysis on the scoring system was then used to define utility in predicting outcome and set cut offs with different sensitivity and specificity. The cut point for identifying high risk patients was determined by Youden’s formula. Goodness of fit of each model was assessed by the Hosmer–Lemeshow chi square test. Finally, repeated 5-fold cross validation was performed to test for internal validation of the scoring system. According to this, the cohort was randomly partitioned into five roughly equal sets; four sets were used to create the model and the other held-out set was used to calculate the prediction error of the fitted model. The same process was repeated for each set and the model’s performance was calculated by averaging the prediction errors across the different test sets [24].

### 2.3. Statistical Power

For characteristics occurring in 50% of the population, this study, with 316 fractures, can detect a difference in prevalence of 15.5%, with 80% power (α = 0.05); when characteristics occur in 10% of the population, a difference in prevalence of 26.1% can be detected by this study, with 80% power (α = 0.05). This being the case, only differences in the occurrence of characteristics of more than 10% were considered.

## 3. Results

### 3.1. Descriptive Statistics

Out of 561 subtrochanteric fractures identified, 84 fractures failed to unite (incidence 15.0%). Following exclusion of the bilateral fractures (only the first fracture was considered), deceased patients (before fracture consolidation), and patients with inadequate follow-up/incomplete clinical or radiological data, 316 patients were included in our final analysis (232 unions; 84 non-unions). Atrophic non-unions were the commonest (67 fractures; 78.8%), followed by hypertrophic non-unions (12 fractures; 14.1%), and septic non-unions (6 fractures; 7.1%). There was no significant difference between the different fracture patterns as per Russell Taylor classification. The average age at the time of the index procedure was 69.13 y.o. (SD 20.01 y.o.), with 126 patients (39.9%) being male (Table 1). The commonest mode of injury was falls from standing height (237 patients; 75.0%), followed by high energy injuries such as road traffic collisions (65 patients; 20.6%) and pathological fractures (14 patients; 0.4%).

### 3.2. Univariate Analysis

In a preliminary unadjusted analysis (not adjusting for cofounders), several factors were identified, being significantly associated with progression of a subtrochanteric fracture to a non-union (Table 2). Age (*p* = 0.610), gender (*p* = 0.999) and mechanism of injury (*p* = 0.115) had no association with progression to non-union. Failure at lag screw junction (metalwork breakage) had the highest unadjusted odds for progressing to non-union (OR, 87.10; 95% CI, 11.54 to 657.56), followed by deep infection (OR, 29.48; 95% CI, 3.67 to 236.74), cut-out of the lag screw (OR, 14.62; 95% CI, 1.68 to 127.05) and self-dynamisation (OR, 9.87; 95% CI, 3.46 to 28.13), defined as failure/breakage of the distal locking screws.

### 3.3. Multivariate Analysis

The identified variables from the unadjusted analysis were then used to build an adjusted multivariable model, which successfully identified seven factors contributing to the development of a non-union (Table 3A). Failure at lag screw junction was excluded from the model because of the absolute relation between failure of the nail and non-union: that is failure at the lag screw junction always led to non-union (out of the 24 patients presenting with failure at the lag screw junction, one patient was managed conservatively because of declining revision surgery; the fracture eventually healed 26 months post-surgery). From the identified associations, deep infection was the most important, followed by self-dynamisation. Presence of an atypical fracture was also significant in the development of a non-union, as was presence of diabetes (insulin or tablet depended). Finally, malreduction, as demonstrated by a lateral cortex fracture gap size and varus malalignment was also strongly associated with the development of a non-union. On the other hand, moderate comminution (as opposed to single 2-part fracture or multi-segmented fracture) seemed to have a “protective” effect.

As self-dynamisation was generally observed at a later stage (up to six months post-operatively), a second logistic regression analysis that excluded this factor was performed to be able to utilise the scoring system in the early post-operative phase (that is within six weeks following the operation) (Table 3B).

### 3.4. Non-Union Risk Score

**Figure 1:**
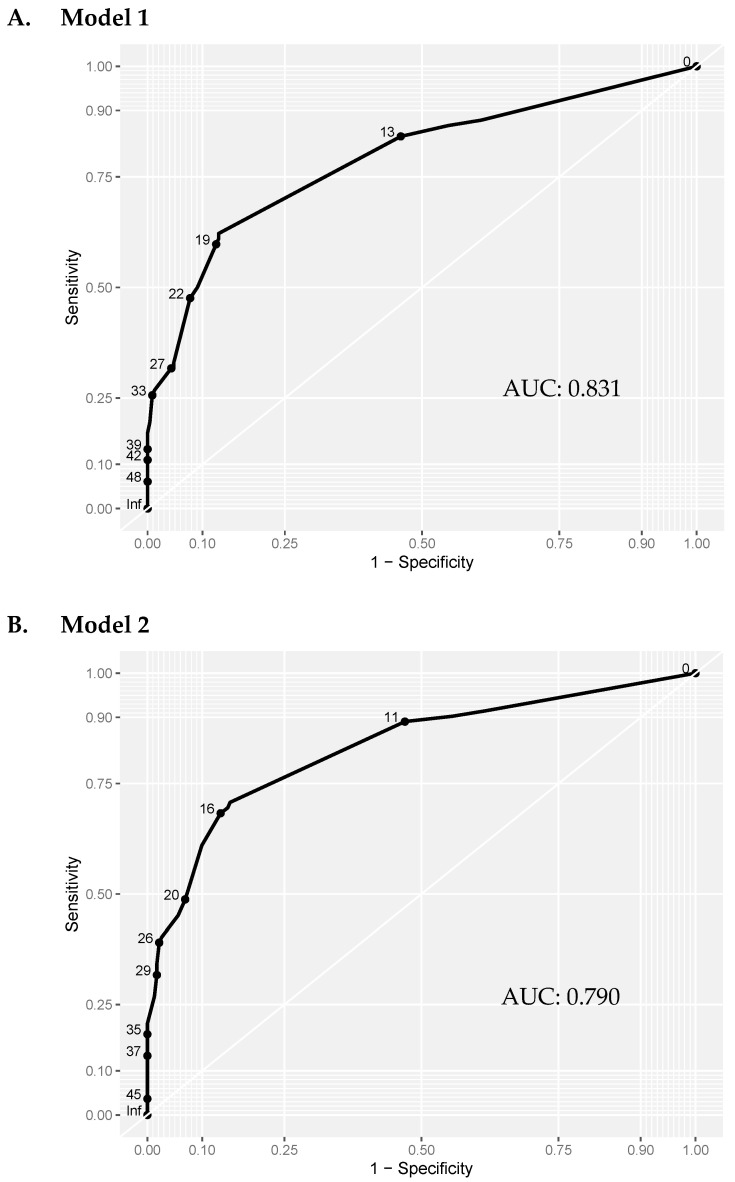
ROC curve analysis of the multivariate prediction models. Model 1: including self-dynamisation. Model 2: excluding self-dynamisation. The discriminatory capability was similar in model 1 (including self-dynamisation; AUC = 0.831), and model 2 (excluding self-dynamisation; AUC = 0.790) was comparable. AUC: Area Under the Curve.

**Figure 2:**
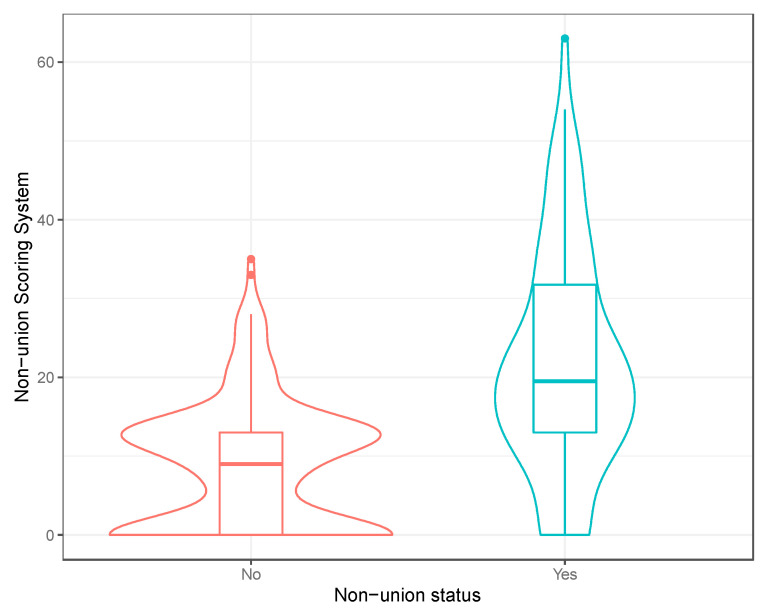
Violin plot of the non-union scoring system (excluding self-dynamisation). A boxplot of the distribution has been included within the violin plot.

Having established the risk factors for non-union, the coefficients were used to produce a risk score for predicting non-union (Table 3A). To test the validity of the scoring system, a ROC curve was produced and the area under the curve (AUC) was calculated. With self-dynamisation included, the discriminatory capability of the multiple logistic regression model (Model 1) was excellent (AUC 0.831) (Figure 1A). To identify the high-risk patients in the early post-operative period, a second scoring system was produced, excluding self-dynamisation (Table 3B). The discriminatory capability of the multiple logistic regression model excluding self-dynamisation (Model 2) remained very good (AUC 0.790) (Figure 1B; Figure 2).

**Figure 3:**
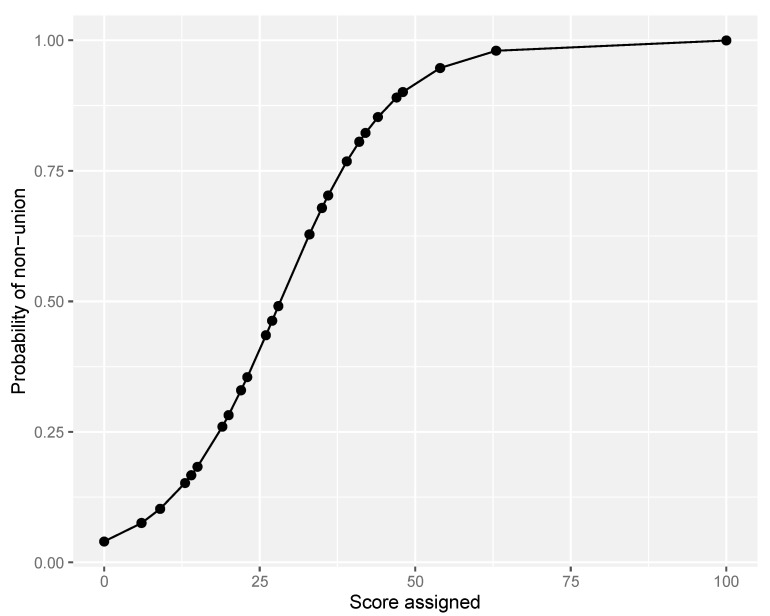
Probability curve of non-union according to score assigned (excluding self-dynamisation).

Because of the advantage of early use of Model 2 (excluding self-dynamisation), we advocate its further use in clinical practice. Furthermore, the goodness of fit of Model 2 (excluding self-dynamisation) was assessed by the Hosmer–Lemeshow test, revealing good calibration of the model (chi-square, 3.744; degrees of freedom = 3; *p* = 0.291). Finally, 5-fold cross validation demonstrated good fit of the model and internal validity (accuracy 0.806; Kappa 0.416). The corresponding probability of the scoring system was then calculated (Table 4; Figure 3), and the cut-point determined by Youden’s formula was calculated as 18.

## 4. Discussion

Non-union remains one of the most debilitating and difficult to treat complications of subtrochanteric fractures. In our series, 84 out of 561 fractures (15.0%) failed to unite. This is comparable to the reported range in the literature (2.3% to 23%) [5,7,25,26,27], whilst differences between studies could possibly be explained by use of different definitions of non-union. Regardless of the incidence reported in each study, the absolute number of non-unions of the subtrochanteric region is increasing along with the increase in the number of proximal femoral fractures worldwide. Reporting associations with progression to non-union can therefore be an important step in the early identification of the high-risk patients and in some cases, even the prevention of this complication. 

We have therefore created and successfully validated a non-union risk score system, which not only identifies the factors associated with a non-union, but also provides a guidance on the modifiable, surgeon related factors, which can be used to further educate surgeons. According to our findings, a score of 18 was determined by Youden’s formula as the cut-point of non-union (excluding self-dynamisation). This implies that patients presenting with even one risk factor (i.e., deep infection or varus malalignment >10 degrees), would be high risk for progressing to non-union. Otherwise, other than the combination of diabetes and varus malalignment of 5–10 degrees, presence of two risk factors would be associated with progression to non-union. Therefore, appropriate counselling could be offered to these high-risk patients, with possible an early intervention. This could range from a minimally invasive procedure (i.e., injection of bone marrow (BM) concentrate), to more aggressive procedures such as bone grafting and revision of the fixation. 

In line with our findings, several authors have suggested deep infection as a causative factor of a non-union [28,29,30]. This could be explained by the ongoing inflammation that disrupts the fracture callus, increases the gap between the fracture site and inadvertently reduces the bone mineral density (BMD) around the affected area, therefore resulting in mechanical instability [28,29,31]. Self-dynamisation on the other hand is another factor associated to a non-union [5], usually followed by a subsequent nail failure proximally [12,32,33]. As self dynamisation usually happens more than six weeks post fixation, by excluding this factor from our analysis we were able to calculate the risk of non-union in the early post-operative period, without any significant reduction in the diagnostic accuracy and validity of our model. 

The presence of an atypical fracture was a further factor contributing to non-unions in our series. Atypical fractures are considered to be secondary to inhibition of osteoclastic bone resorption, which in turn can lead to over-mineralisation that makes the bone more brittle, and accumulation of microdamage that increase the risk of pathological fractures [34,35]. Impaired bone healing following atypical fractures is a common finding in most studies [36,37,38,39,40,41], whilst the incidence of revision for any cause has been reported to be as high as 46% [39,42,43].

Malreduction is another potentially preventable cause of non-union. In our cohort, this was demonstrated by a lateral cortex fracture gap size (more than 5 mm) and a varus malalignment (there was an increase in the risk of non-union with an increase in the varus of fixation). Whilst varus malalignment has been reported as a risk factor for non-unions by a number of authors [5,7,26,44,45], lateral cortex gap size has never been identified as such.

Diabetes, a common chronic metabolic disease, has also been identified as a factor related to an increased risk of non-union. On a cellular level, there is an increase in pro-inflammatory mediators in diabetic patients, whilst the downregulation of inflammation is also reduced [46], leading to enhanced osteoclastogenesis and decreased osteoblastic activity [46,47]. This is further enhanced by the direct effect of insulin [47,48,49] and hyperglycaemia on osteoblasts and osteoclasts [47,50,51]. Additionally, the micro- and macro- angiopathy secondary to diabetes, increases the risk of impaired healing and wound problems [46,52]. In fractures of the lower extremity, several authors have reported a clear association of diabetes and delayed union/non-union [51,53,54].

Finally, it was demonstrated by our cohort that the degree of comminution had a significant contribution to progression to a non-union. More specifically, simple (two fragments) or severe comminution (four fragments or more) was associated with a higher risk compared to moderate comminution (three fracture fragments). In the literature, only lack of medial cortical support (i.e., medial cortical comminution), has been reported as a risk factor for subtrochanteric non-union [5]. The association of the higher degree of comminution and impaired fracture healing may be secondary to the disruption of the blood supply of the fragments, as well as the subsequent instability at the fracture site. With regards to the ‘simple’ two-part subtrochanteric fractures, their increased risk of non-union may be secondary to the high incidence of malreduction of these complex fractures, as well as their high association with atypical fractures, which was also demonstrated to be a risk factor for a non-union in our series.

The study strengths include the comprehensive interrogation and cross examination of the health records of each patient to ensure accuracy of data collected, along with the independent, blinded evaluation of all the radiological parameters. The broad inclusion criteria provide a better representation of a large metropolitan population covered by a single Level 1 Trauma Centre. Limitations of the study include its retrospective nature and loss of follow-up to a number of patients, either because of mortality, follow-up in other institutions or non-attendance. Should the loss to follow up not be related to the outcome (union or non-union), which we anticipate is the case, then our findings hold. Finally, one could argue that the results from a single institution may not be applicable in other centres, but the large and diverse sample reduces this risk.

## 5. Conclusions

This study demonstrates that the risk of non-union can be reliably estimated in patients presenting with a subtrochanteric fracture, from the immediate post-operative period. The resulting non-union risk score is based on wound infection, presence of diabetes, original fracture characteristics (atypical fractures and presence of severe comminution), and most importantly surgery related factors (presence of a lateral cortex gap and varus malreduction of the fracture). By identifying these patients early, appropriate consultation and in some cases surgical intervention could be offered, therefore reducing the overall time to union and all the direct and indirect costs resulting from this devastating complication, transforming the care of these fracture patients. At the same time, surgeons should try to avoid all the modifiable surgery related factors that increase this risk, whilst aggressive management of these factors is advocated (i.e., early aggressive management of wound infections, improved diabetic control and referral to endocrinology in cases of atypical fractures).

## Figures and Tables

**Table 1:** Table presenting the demographics/characteristics of patients having their operation in our institution, with complete follow-up, stratified according to the progression to a non-union.

**Demographics**		**All Patients**	**Union**	**Non-Union**
N		316	232 (73.4%)	84 (24.6%)
Age (y.o.)		69.13 ± 20.01	69.48 ± 20.81	68.18 ± 17.70
Gender	Male	126 (39.9%)	92 (39.7%)	34 (40.5%)
	Female	190 (60.1%)	140 (60.3%)	50 (59.5%)
**Injury Characteristics**		**All Patients**	**Union**	**Non-Union**
Mechanism of Injury	Low energy	237 (75.0%)	178 (76.7%)	59 (70.2%)
	High energy	65 (20.6%)	47 (20.3%)	18 (21.4%)
	Pathological	14 (0.4%)	7 (3.0%)	7 (8.3%)
Isolated		264 (83.5%)	191 (82.3%)	73 (86.9%)
ISS > 16		25 (7.9%)	17 (7.3%)	8 (9.5%)
Side	Left	161 (50.9%)	116 (50.0%)	45 (53.6%)
	Right	155 (49.1%)	116 (50.0%)	39 (46.4%)
Open fracture		7 (2.2%)	4 (1.7%)	3 (3.6%)
**Medical Comorbidities**		**All Patients**	**Union**	**Non-Union**
ASA	1	40 (12.7%)	35 (15.1%)	5 (6.0%)
	2	92 (29.1%)	64 (27.6%)	28 (33.3%)
	3	149 (47.2%)	107 (46.1%)	42 (50.0%)
	4	35 (11.0%)	26 (11.2%)	9 (10.7%)
Charlson Comorbidity Score		4.614 ± 3.04	4.56 ± 3.03	4.76 ± 3.06
Diabetes		42 (13.3%)	25 (10.8%)	17 (20.2%)
Steroids		14 (4.4%)	10 (4.3%)	4 (4.8%)
Malignancy		69 (21.8%)	48 (20.7%)	21 (25.0%)
Dementia		39 (12.3%)	34 (14.7%)	5 (6.0%)
**Osteoporosis**		**All Patients**	**Union**	**Non-Union**
Bisphosphonates pre-admission		60 (19.0%)	40 (17.2%)	20 (23.8%)
Bisphosphonates on discharge		86 (27.4%)	63 (27.2%)	23 (28.0%)
Calcium/Vitamin D pre-admission		83 (26.3%)	58 (25.0%)	25 (29.8%)
Calcium/Vitamin D on discharge		142 (45.2%)	103 (44.4%)	39 (47.6%)
Vitamin D loading on admission		42 (13.4%)	34 (14.7%)	8 (9.8%)
Fragility Fractures Before		56 (17.8%)	40 (17.2%)	16 (19.3%)
Fragility Fractures After		62 (19.9%)	44 (19.0%)	18 (21.7%)
DEXA Result	Normal	5 (12.5%)	3 (10.3%)	2 (18.2%)
	Osteopenia	13 (32.5%)	7 (24.1%)	6 (54.5%)
	Osteoporosis	22 (55.0%)	19 (65.5%)	3 (27.3%)
Singh Index	1	24 (8.4%)	18 (8.5%)	6 (8.2%)
	2	61 (21.4%)	48 (22.6%)	13 (17.8%)
	3	56 (19.6%)	41 (19.3%)	15 (20.5%)
	4	66 (23.2%)	48 (22.6%)	18 (24.7%)
	5	33 (11.6%)	22 (10.4%)	11 (15.1%)
	6	45 (15.8%)	35 (16.5%)	10 (13.7%)
**Social History**		**All Patients**	**Union**	**Non-Union**
Smoking		68 (21.5%)	49 (21.1%)	19 (22.6%)
Alcohol > 10 units/week		67 (21.2%)	44 (19.0%)	23 (27.4%)
Pre-operative Mobility				
Independent		174 (55.1%)	129 (55.6%)	45 (53.6%)
Stick(s)/Crutch(es)		94 (29.7%)	62 (26.7%)	32 (38.1%)
Frame		35 (11.1%)	30 (12.9%)	5 (6.0%)
Wheelchair/Hoisted		13 (4.1%)	11 (4.7%)	2 (2.4%)
Frequent falls		80 (25.3%)	61 (26.3%)	19 (22.6%)
**Operation Characteristics**		**All Patients**	**Union**	**Non-Union**
Operation in less than 48 h		247 (78.2%)	182 (78.4%)	65 (77.4%)
Simultaneous procedures		27 (8.5%)	22 (9.5%)	5 (6.0%)
Type of Nail	Long Affixus Nail	160 (50.6%)	124 (53.4%)	36 (42.9%)
	Long Gamma Nail	128 (40.5%)	90 (38.8%)	38 (45.2%)
	Others	28 (8.9%)	18 (7.8%)	10 (11.9%)
Nail Diameter	9	18 (5.8%)	9 (3.9%)	9 (10.8%)
(mm)	10	7 (2.1%)	4 (1.7%)	3 (3.6%)
	11	203 (64.9%)	154 (67.0%)	49 (59.0%)
	13	85 (27.2%)	63 (27.4%)	22 (26.5%)
Open reduction		151 (47.8%)	104 (44.8%)	47 (56.0%)
Use of cerclage wires		39 (12.3%)	33 (14.2%)	6 (7.1%)
Post-op Mobilisation	FWB	148 (46.9%)	113 (48.7%)	35 (41.7%)
(first 6 weeks)	PWB	80 (25.3%)	58 (25.0%)	22 (26.2%)
	TTWB	51 (16.1%)	41 (17.7%)	10 (11.9%)
	NWB	37 (11.7%)	20 (8.6%)	17 (20.2%)
Surgical time (min)		113.11 ± 45.56	111.32 ± 45.50	118.2 ± 45.62
Anaesthetic Time (min)		47.66 ± 22.82	47.22 ± 22.76	48.91 ± 23.08
Time from induction to recovery (min)		179.94 ± 50.26	177.57 ± 49.40	186.63 ± 52.34
Level of First Surgeon				
	Registrar	193 (61.5%)	142 (61.2%)	51 (62.2%)
	Consultant	121 (38.5%)	90 (38.8%)	31 (37.8%)
Level of Senior Surgeon Present				
	Registrar	178 (56.7%)	131 (56.5%)	47 (57.3%)
	Consultant	136 (43.3%)	101 (43.5%)	35 (42.7%)
**Complications**		**All Patients**	**Union**	**Non-Union**
Nail complications		78 (24.7%)	34 (14.7%)	44 (52.4%)
Failure at lag screw junction		24 (7.6%)	1 (0.4%)	23 (27.4%)
Self-dynamisation		20 (6.3%)	5 (2.2%)	15 (17.9%)
Cut-out		6 (1.9%)	1 (0.4%)	5 (6.0%)
Nail infection		5 (1.6%)	3 (1.3%)	2 (2.4%)
Peri-implant fracture		8 (2.5%)	7 (3.0%)	1 (1.2%)
HAP/CAP		46 (14.6%)	35 (15.1%)	11 (13.1%)
UTI		45 (14.2%)	35 (15.1%)	10 (11.9%)
Wound infection	Superficial	11 (3.5%)	5 (2.2%)	6 (7.1%)
	Deep	10 (3.2%)	1 (0.4%)	9 (10.7%)
Washout/Revision for Infection		6 (8.2%)	2 (10.5%)	4 (7.4%)
CKD Stage pre-operatively				
	Mild	220 (71.2%)	169 (74.4%)	51 (62.2%)
	Moderate/Severe	89 (28.8%)	58 (25.6%)	31 (37.8%)
CKD Stage post-operatively				
	Mild	227 (74.4%)	170 (76.2%)	57 (69.5%)
	Moderate/Severe	78 (25.6%)	53 (23.8%)	25 (30.5%)
Pre-operative Transfusion		25 (7.9%)	21 (9.1%)	4 (4.8%)
Post-operative Transfusion (48 h)		153 (48.6%)	111 (47.8%)	42 (50.6%)
Post-operative Transfusion (total)		192 (61.0%)	138 (59.5%)	54 (65.1%)
Hb Drop (g/L)		44.29 ± 18.24	44.13 ± 18.30	44.72 ± 18.20
**Biochemistry**		**All Patients**	**Union**	**Non-Union**
Adjusted Calcium	Normal	181 (74.8%)	141 (79.7%)	40 (61.5%)
	Low	61 (25.2%)	36 (20.3%)	25 (38.5%)
Albumin	Normal	106 (38.4%)	79 (38.7%)	27 (37.5%)
	Low	170 (61.6%)	125 (61.3%)	45 (62.5%)
Alkaline Phosphatase High		55 (20.1%)	40 (19.9%)	15 (20.8%)
	Normal	201 (73.7%)	149 (74.1%)	52 (72.2%)
	Low	17 (6.2%)	12 (6.0%)	5 (6.9%)
Phosphate Normal/High		201 (82.4%)	148 (83.1%)	53 (80.3%)
	Low	43 (17.6%)	30 (16.9%)	13 (19.7%)
TSH	High	13 (9.2%)	9 (8.5%)	4 (11.4%)
	Normal	126 (89.4%)	95 (89.6%)	31 (88.6%)
	Low	2 (1.4%)	2 (1.9%)	0 (0.0%)
Free T4	High	20 (14.4%)	17 (16.2%)	3 (8.8%)
	Normal	116 (83.5%)	85 (81.0%)	31 (91.2%)
	Low	3 (2.1%)	3 (2.9%)	0 (0.0%)
PTH	High	62 (48.8%)	47 (53.4%)	15 (38.5%)
	Normal	65 (51.2%)	41 (46.6%)	24 (61.5%)
Total 25OH Vitamin D Normal		17 (12.1%)	13 (12.7%)	4 (10.3%)
	Low	124 (87.9%)	89 (87.3%)	35 (89.7%)
**Radiographic Measurements**		**All Patients**	**Union**	**Non-Union**
Femoral Neck Shaft Angle				
	Normal	209 (67.4%)	150 (65.8%)	59 (72.0%)
	Coxa Valga	89 (28.7%)	70 (30.7%)	19 (23.2%)
	Coxa Vara	12 (3.9%)	8 (3.5%)	4 (4.9%)
Number of fragments	Simple	88 (28.0%)	58 (25.0%)	30 (36.6%)
(Comminution)	Moderate	153 (48.8%)	131 (56.5%)	22 (26.8%)
	Severe	73 (23.2%)	43 (18.5%)	30 (36.6%)
Isolated Subtrochanteric Extension		49 (15.6%)	33 (14.2%)	16 (19.5%)
Atypical		20 (6.4%)	7 (3.0%)	13 (15.9%)
Pathological		11 (3.5%)	7 (3.0%)	4 (4.9%)
Distal Extension		123 (39.2%)	91 (39.2%)	32 (39.0%)
Lesser Trochanter Fracture		203 (64.6%)	154 (66.4%)	49 (59.8%)
Medial Calcar Comminution		21 (6.7%)	16 (6.9%)	5 (6.1%)
Lateral Cortex Gap Size	≤4	191 (60.4%)	159 (68.5%)	32 (38.1%)
(mm)	5–9	85 (26.9%)	48 (20.7%)	37 (44.0%)
	≥10	40 (12.7%)	25 (10.8%)	15 (17.9%)
Medial Cortex Gap Size	≤4	210 (66.5%)	166 (71.6%)	44 (52.4%)
(mm)	5–9	72 (22.8%)	43 (18.5%)	29 (34.5%)
	≥10	34 (10.7%)	23 (9.9%)	11 (13.1%)
Anterior Cortex Gap Size	≤4	201 (63.6%)	156 (67.2%)	45 (53.6%)
(mm)	5–9	68 (21.5%)	48 (20.7%)	20 (23.8%)
	≥10	47 (14.9%)	28 (12.1%)	19 (22.6%)
Posterior Cortex Gap Size	≤4	231 (73.1%)	185 (79.7%)	46 (54.8%)
(mm)	5–9	64 (20.2%)	34 (14.7%)	30 (35.7%)
	≥10	21 (6.7%)	13 (5.6%)	8 (9.5%)
Reduction Angle Grouped				
(degrees)	Valgus 5–Varus 5	233 (73.7%)	188 (81.0%)	45 (53.6%)
	Valgus >5	17 (5.4%)	10 (4.3%)	7 (8.3%)
	Varus 5–10	52 (16.5%)	29 (12.5%)	23 (27.4%)
	Varus >10	14 (4.4%)	5 (2.2%)	9 (10.7%)
Anti-rotation Screw		110 (35.6%)	84 (37.0%)	26 (31.7%)
TAD	<25	259 (84.6%)	193 (86.2%)	66 (80.5%)
(mm)	≥25	47 (15.4%)	31 (13.8%)	16 (19.5%)
Distal locking	1	10 (3.2%)	9 (3.9%)	1 (1.2%)
(Number of Screws)	2	306 (96.8%)	223 (96.1%)	83 (98.8%)
Method of locking				
Static Locking		204 (64.8%)	153 (66.2%)	51 (60.7%)
Secondary Dynamisation		108 (34.3%)	75 (32.5%)	33 (39.3%)
Dynamic		3 (0.9%)	3 (1.3%)	0 (0.0%)
Distance of tip of the nail from centre (AP)				
(mm)	−4 to 4	200 (63.7%)	153 (66.5%)	47 (56.0%)
	Lateral ≥5	64 (20.4%)	41 (17.8%)	23 (27.4%)
	Medial ≥5	50 (15.9%)	36 (15.7%)	14 (16.7%)
Distance of tip of the nail from centre (LAT) (mm)				
	−4 to 4	256 (81.5%)	186 (80.9%)	70 (83.3%)
	Anterior ≥5	53 (16.9%)	41 (17.8%)	12 (14.3%)
	Posterior ≥5	5 (1.6%)	3 (1.3%)	2 (2.4%)
Distance of tip of the nail from knee				
(mm)	<10	2 (0.6%)	2 (0.9%)	0 (0.0%)
	10 to 19	24 (7.6%)	13 (5.7%)	11 (13.1%)
	20–29	99 (31.5%)	78 (33.9%)	21 (25.0%)
	≥30	189 (60.3%)	137 (59.6%)	52 (61.9%)
Nail/Canal Ratio		0.82 ± 0.08	0.82 ± 0.08	0.83 ± 0.07
**Hospital Stay**		**All Patients**	**Union**	**Non-Union**
HDU/ICU stay		36 (11.4%)	21 (9.1%)	15 (17.9%)
Total length of hospital stay (days)		21.26 ± 19.19	20.74 ± 18.00	22.69 ± 22.22
Weekend admission		105 (33.2%)	76 (32.8%)	29 (34.5%)

Dichotomous variables are presented as absolute numbers (percentages) of the positive event. Continuous variables are presented as mean (SD). ISS: Injury Severity Score; ASA: American Society of Anaesthesiologists Classification; DEXA: Dual-energy X-ray absorptiometry; FWB: Full Weight Bearing; PWB: Partial Weight Bearing; TTWB: Toe-touch weight bearing; NWB: Non-weight bearing; HAP: Hospital Acquired Pneumonia; CAP: Community Acquired Pneumonia; UTI: Urinary Tract Infection; CKD: Chronic Kidney Disease; DVT: Deep Vein Thrombosis; VTE: Venous Thromboembolism; TAD: Tip Apex distance; AP: Anterior-Posterior view; LAT: Lateral view; HDU: High Dependency Unit; ICU: Intensive Care Unit.

**Table 2:** Unadjusted associations with progression to non-union.

**Medical Comorbidities**		**Unadjusted OR** **(95% CI)**	***p*-Value**
Diabetes		2.10 (1.07–4.13)	0.031
**Operation Characteristics**		**Unadjusted OR ** **(95% CI)**	***p*-Value**
Post-op Mobilisation	FWB	Ref	Ref
(first 6 weeks)	PWB	1.23 (0.66–2.28)	0.522
	TTWB	0.79 (0.358–1.73)	0.553
	NWB	2.74 (1.30–5.81)	0.008
**Complications**		**Unadjusted OR ** **(95% CI)**	***p*-Value**
Nail complications		6.41 (3.65–1.24)	<0.001
Failure at lag screw junction		87.10 (11.54–657.56)	<0.001
Self-dynamisation		9.87 (3.46–28.13)	<0.001
Cut-out		14.62 (1.68–127.05)	0.015
Wound infection	Superficial	3.93 (1.16–13.27)	0.028
	Deep	29.48 (3.67–236.74)	0.001
CKD Stage pre-operatively			
	Mild	Ref	Ref
	Moderate/Severe	1.77 (1.04–3.03)	0.037
**Biochemistry**		**Unadjusted OR ** **(95% CI)**	***p*-Value**
Adjusted Calcium	Normal	Ref	Ref
	Low	2.45 (1.32–4.55)	0.005
**Radiographic Measurements**		**Unadjusted OR ** **(95% CI)**	***p*-Value**
Number of fragments			
(Comminution)	Moderate	Ref	Ref
	Simple -Severe	0.28 (0.16–0.49)	<0.001
Atypical		6.06 (2.32–15.78)	<0.001
Lateral Cortex Gap Size	≤4	Ref	Ref
(mm)	≥5	3.54 (2.10–5.96)	<0.001
Medial Cortex Gap Size	≤4	Ref	Ref
(mm)	5–9	2.54 (1.43–4.53)	0.001
	≥10	1.80 (0.82–3.98)	0.144
Anterior Cortex Gap Size	≤4	Ref	Ref
(mm)	5–9	1.44 (0.78–2.68)	0.244
	≥10	2.35 (1.20–4.60)	0.012
Posterior Cortex Gap Size	≤4	Ref	Ref
(mm)	5–9	3.55 (1.97–6.39)	<0.001
	≥10	2.48 (0.97–6.32)	0.058
Reduction Angle Grouped			
(degrees)	Varus <5	Ref	Ref
	Varus 5–10	3.02 (1.61–5.65)	0.001
	Varus >10	6.85 (2.20–21.33)	0.001
**Hospital Stay**			***p*-Value**
HDU/ICU stay		2.18 (1.07–4.47)	0.033

OR: Odds Ratio; CI: Confidence Interval; FWB: Full Weight Bearing; PWB: Partial Weight Bearing; TTWB: Toe-touch weight bearing; NWB: Non-weight bearing; CKD: Chronic Kidney Disease; Ref: Reference.

**Table 3:** Multivariate prediction models of non-union risk following a subtrochanteric fracture.

A Model 1: Associations with progression to non-union, including self-dynamisation; OR: Odds Ratio					
**Model 1**	**Score**	**β Coefficient**	**Standard Error**	**Adjusted OR (95% CI)**	***p*** **-Value**
Diabetes	5	0.79	0.45	2.20 (0.92–5.25)	0.077
Self-dynamisation	20	3.03	0.63	20.74 (6.09–70.68)	<0.001
Wound infection (Deep)	29	4.35	1.14	77.80 (8.26–732.71)	<0.001
Degree of comminution (Simple or Severe)	11	1.62	0.37	5.05 (2.44–10.46)	<0.001
Atypical	11	1.59	0.58	4.92 (1.58–15.37)	0.006
Lateral Cortex Gap Size (≥5 mm)	10	1.44	0.36	4.24 (2.12–8.50)	<0.001
Reduction Angle (Varus 5–10 degrees)	7	1.01	0.42	2.75 (1.21–6.23)	0.016
Reduction Angle (Varus >10 degrees)	15	2.34	0.71	10.33 (2.59–41.26)	0.001
B Model 2: Associations witd progression to non-union, not including self-dynamisation; OR: Odds Ratio					
**Model 2**	**Score**	**β Coefficient**	**Standard Error**	**Adjusted OR (95% CI)**	***p*-Value**
Diabetes	6	0.70	0.42	2.02 (0.88–4.63)	0.096
Wound infection (Deep)	35	3.97	1.12	53.05 (5.87–479.64)	<0.001
Degree of comminution (Simple or Severe)	13	1.50	0.34	4.47 (2.29–8.74)	<0.001
Atypical	14	1.53	0.55	4.63 (1.58–13.56)	0.005
Lateral Cortex Gap Size (≥5 mm)	11	1.21	0.32	3.37 (1.78–6.36)	<0.001
Reduction Angle (Varus 5–10 degrees)	9	1.02	0.39	2.76 (1.30–5.88)	0.008
Reduction Angle (Varus >10 degrees)	20	2.27	0.70	9.71 (2.51–37.49)	0.001

**Table 4:** Non-union risk score and corresponding probability of non-union.

Non-Union Risk Score	Probability of Non-Union
0	4.0%
6	7.6%
9	10.3%
13	15.2%
14	16.7%
15	18.3%
19	26.0%
20	28.2%
22	33.0%
23	35.5%
26	43.5%
27	46.3%
28	49.1%
33	62.8%
35	67.9%
36	70.3%
39	76.8%
41	80.6%
42	82.3%
44	85.3%
47	89.0%
48	90.1%
54	94.7%
63	98.0%
100 *	100.0%

* Extrapolated value. A score of 100 was not observed in any of the patients.

## Data Availability

The data presented in this study are available on reasonable request from the corresponding author (M.P.).
